# Supplementary figures and images for: Escherichia coli Producing Extended-Spectrum β-lactamases (ESBL) from Domestic Camels in the Canary Islands: A One Health Approach
Source: Animals (Basel). 2020 Jul 29;10(8):1295. doi: 10.3390/ani10081295 (PMC7459641; doi:10.3390/ani10081295)

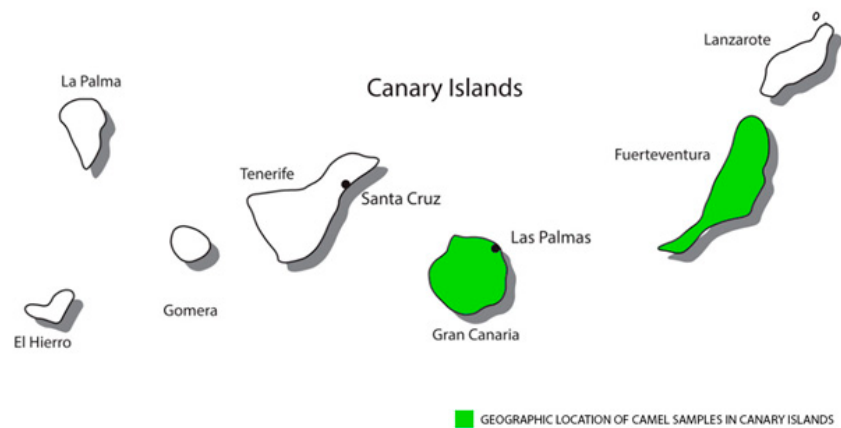

**Figure S1** – Origin of camels' samples: Fuerteventura and Gran Canaria (Canary Islands, Spain).

Supplement: Supplementary file 1 [file animals-10-01295-s001.pdf]
